# Experimental Study on Mechanical and Sensing Properties of Smart Composite Prestressed Tendon

**DOI:** 10.3390/ma11112087

**Published:** 2018-10-25

**Authors:** Danhui Dan, Pengfei Jia, Guoqiang Li, Po Niu

**Affiliations:** 1 Key Laboratory of Performance Evolution and Control for Engineering Structures of Ministry of Education, Tongji University, Shanghai 200092, China; 2 Department of Bridge Engineering, Tongji University, Shanghai 200092, China; jiapengfei@tongji.edu.cn; 3 China Railway Engineering Consulting Group Co., Ltd., Beijing 100055, China; lgqbj@sohu.com; 4 Zhenshixing Technology Co., Ltd., Tianjin 300384, China; s15122675688@163.com

**Keywords:** carbon fiber bar, Fiber Bragg Grating, strain sensor, smart composite prestressed tendon, experimental study

## Abstract

It is typically difficult for engineers to detect the tension force of prestressed tendons in concrete structures. In this study, a smart bar is fabricated by embedding a Fiber Bragg Grating (FBG) in conjunction with its communication fiber into a composite bar surrounded by carbon fibers. Subsequently, a smart composite cable is twisted by using six outer steel wires and the smart bar. Given the embedded FBG, the proposed composite cable simultaneously provides two functions, namely withstanding tension force and self-sensing the stress state. It can be potentially used as an alternative to a prestressing reinforcement tendon for prestressed concrete (PC), and thereby provide a solution to detecting the stress state of the prestressing reinforcement tendons during construction and operation. In the study, both the mechanical properties and sensing performance of the proposed composite cable are investigated by experimental studies under different force standing conditions. These conditions are similar to those of ordinary prestressed tendons of a real PC components in service or in a construction stage. The results indicate that the proposed smart composite cable under the action of ultra-high pretension stress exhibits reliable mechanical performance and sensing performance, and can be used as a prestressed tendon in prestressed concrete structures.

## 1. Introduction

The cable is an important component in civil engineering structures, and typically appears in two forms. The first is where most of the cable body is free and only supported or connected with other structural components at its ends or limited points in the middle part (such as cables in a cable-stayed bridge). The second is where a complete cable is connected and contacted everywhere within its supported structure (such as the tendons used in bonded prestressed concrete structures). With respect to both forms, cable materials have gradually changed from their original pig iron into ordinary steel and high-strength steel, and currently involve the use of lighter and stronger fiber materials. Given the effects of the environment, load, corrosion, and fatigue, cable damage gradually accumulates and mechanical properties are gradually degraded. This eventually jeopardizes the health and safety of the entire structure. Thus, mechanical properties of a cable should be monitored to ensure safe and durable structures. The performance of cables in a cable-stayed bridge can be monitored by vibration method, which entails using accelerometers to record the acceleration response of a cable, and identifying cable forces and other physical parameters [1]. Additionally, sensors based on magnetoelasticity [2,3] and acoustic emission technology [4] can also be used to monitor the internal force or stress of a cable. However, an effective monitoring method is absent with respect to prestressed tendons inside prestressed reinforced concrete structures. Thus, a smart cable that includes a sensing element is proposed to replace an ordinary prestressing tendon. The aforementioned studies mainly focus on a cable based on Fiber Bragg Grating (FBG) sensing technology [5].

A FBG based smart cable combines a FBG sensor with steel wires such that it senses its physical and mechanical state in real time. In 1993, Alavie et al. [6] embedded FBG sensors in a carbon fiber prestressed bar on a highway bridge for the first time, realized monitoring of the strain, and subsequently designed and constructed the corresponding monitoring system. However, the production and installation of the sensor is extremely complicated, and it should be connected after the prestressing tendons are stretched in order to reduce the tensile strain range. The actual sensing function is relatively simple. In 2000, Chan et al. [7] placed FBG sensors on the exterior of a rectangular concrete beam to monitor the strain of the beam. However, placing the sensor outside the structure only obtains information outside the structure and not information related to the interior of the concrete and cable. Zhang et al. [8] applied FRP (Fiber Reinforced Plastics) smart bars encapsulated with fiber gratings to the monitoring project in Shenzhen Vanke Center and verified high linearity. This corresponds to an application of a smart cable as an independent component in structures. Zhao et al. [9] conducted an experiment to examine the performance of a smart cable in terms of durability and reliability, and the results indicated that the smart cable still maintained high accuracy after experiencing sufficient fatigue loading. In 2003, Z. Zhou et al. [10] combined FBG and FRP materials to form a smart FRP-OFBG (Optical Fiber Bragg Grating) bar wherein the maximum strain of the bar during the test was only 1200 με. In 2005, Z. Zhou et al. [11] used the same materials to fabricate a smart cable and tested it in a laboratory wherein the maximum strain reached 3400 με. Li et al. [12] repeated the staged tensioning process for GFRP-OFBG smart bars and observed their repeatability and linearity and verified the application of a GFRP (Glass Fiber Reinforced Plastics)-OFBG smart cable structure in steel strands and it can accurately trace an entire tensioning procedure. Yang et al. [13] performed an in-depth examination of the packaging and installation technology of a sensor in concrete and reliability of strain FBG sensors in a concrete structure. In 2011, LI Hui et al. [14] summarized the development of a smart cable that was combined with a steel rebar and fixed with the steel rebars to form a self-sensing smart stay cable. However, each smart cable only included one sensor. ST Kim et al. [15] embedded a Fiber Bragg Grating sensor in the core wire of the PC strand and the experiment found that the measured data appeared to provide sufficient reliability. This paper focuses more on the manufacturing process of smart cables and the physical properties of light. K Cho et al. [16] derived the nonlinear stress-strain model of this strand made of two materials and then [17] proposed a method estimating the final prestress force distribution in individual tendons of a PSC (Prestressed Concrete) girder using the smart strand. Additionally, the industry specification for a “carbon fiber composite cable for bridges” prepared by the Ministry of Transport of the People’s Republic of China is already under review to further standardize the use of this type of a smart cable in real projects.

Despite progress in the research and application of smart cables, extant studies exhibit the following deficiencies: (1) The primary function of a smart cable used in prestressed concrete corresponds to the bearing-load components, and this is followed by the sensing function. Extant studies focus on its sensing performance as opposed to its main stress function. (2) With respect to its use in prestressed tendons, the FBG-based smart cable establishes a high-stress state during construction and holds on in the service stage, and the live load generates stress only at a relatively low increment in a high-stress level during the operation time. This is identical to that of a smart bar. To the best of the authors’ knowledge, all extant studies focus on the sensing performance of a smart cable in the absence of a high-stress working starting point and do not examine the sensing performance of a sensing element (FBG) that functions at a high-strain state. (3) In industrialization construction, in order to obtain construction efficiency, it is typically desirable to tension a smart cable with a simple process such as tensioning the ordinary prestressed tendons. Previous studies do not investigate the effect of the traditional tensioning process on the sensing performance of a smart cable.

In theory, smart composite prestressed tendons correspond to new types of structural members that combine the functions of reinforcement and sensing, and thereby solve the issue related to the prestressing of the components. However, prior to the actual practical phase of the study, it is also necessary for the proposed smart composite prestressed tendons to compensate for the lack of studies in the three aforementioned aspects to solve engineering applicability issues. Therefore, the present study involves the use of a real cable test to explore the performance of a smart tendon under the working conditions of prestressed tendons to lay a practical engineering foundation.

## 2. Smart Composite Prestressed Tendon Solution

The proposed smart composite cable that is used as the prestressed tendon in the prestressed concrete structure is termed a smart composite prestressed tendon. As mentioned previously, the smart tendon generally corresponds to a composite cable structure comprised of a smart bar and ordinary metal wires. The aforementioned types of solutions stress its mechanical properties and also its sensing function to monitor the stress state of the tendon itself. We focus on a solution in which the proposed prestressed smart composite tendon is composed of an inner smart core and outer high strength steel wires on the cross section. The smart core is fabricated by embedding an FBG sensor in a CFRP (Carbon Fiber Reinforced Polymer) bar. The outer steel wires serve as the main bearing function, and the core smart bar provides the sensing function.

### 2.1. Carbon Fiber-Based Smart Bar

**Figure 1:**
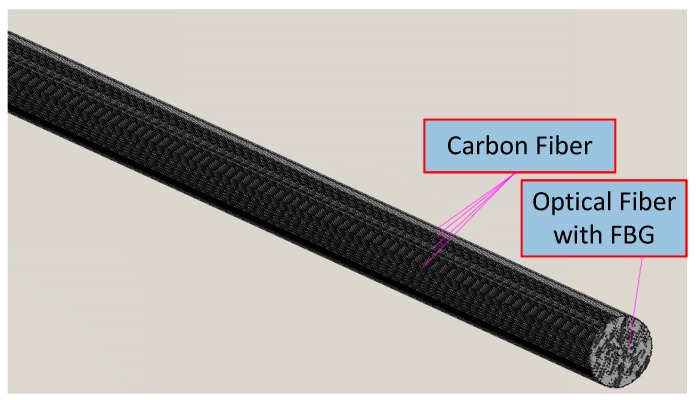
The structure of a carbon fiber smart bar.

A carbon fiber-based smart bar is a sensing component in smart composite prestressed tendons. Although the fiber grating effectively measures the changes in strain, it cannot be solely connected with the structure because it is fragile and does not effectively bond with the steel bar. Carbon fiber is a lightweight fiber material with high-strength, high-corrosion resistance, high-temperature resistance, and low-thermal expansion. Its diameter ranges from 5 to 8 µm, and thus it can be fabricated into a variety of shapes and is extremely suitable for combination with other materials to form composite materials. The tensile strength of the smart bar is 2400 MPa, and the elastic modulus is 160 GPa. The coefficient of thermal expansion (CTE) of the smart bar is 0.078 × 10^−6^ (1/°C) for about 20 °C. The Bragg sensitivity is 1.21 pm/microstrain (at 1550 nm). In the study, the FBG bar and carbon fibers are combined to form a carbon fiber-based smart bar that exhibits both the aforementioned advantages. The structure of the smart bar is shown in Figure 1.

The smart bars are produced by the pultrusion of a carbon fiber and FBG bars in resin glue such that it forms a complete bar. In the production process, it is necessary to ensure the survival of the FBG bar and to ensure that the FBG bar is evenly distributed along the smart bar. The diameter of the smart bar is identical as that of the ordinary steel wire, and thus that the composite smart bar is constructed based on the normal construction process. The length of the smart bar should satisfy the needs of different projects. The smart bars produced based on the aforementioned method can be independently placed in the structure as a component, or they can be combined with several steel wires into strands to form a composite cable. Additional composite cables can be combined to form a bridge cable or a hanger. They can also be used to replace traditional prestressed steel strands or tendons in prestressed concrete to form a smart composite prestressed tendon as mentioned in following section.

### 2.2. Smart Composite Prestressed Tendon

**Figure 2:**
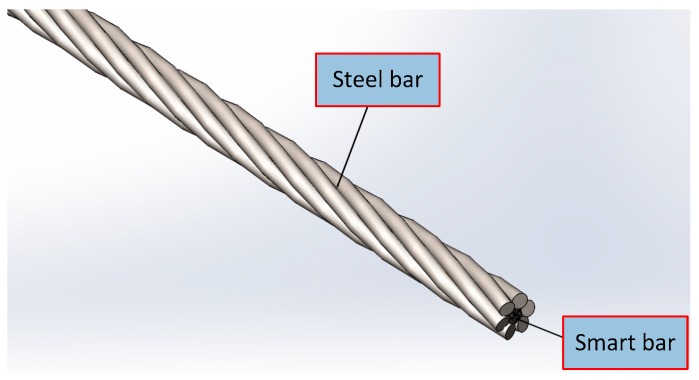
Schematic diagram of a smart composite prestressed tendon.

First, the seven steel wires are wound into bundles according to the process of making steel strands. Then the steel wire located at center is then withdrawn and replaced with the aforementioned carbon fiber-based smart bar. Finally, the steel bars and the smart bar are again wound to become one. Subsequently, a smart composite prestressed tendon is fabricated. The schematic diagram of a prestressed smart composite cable is shown in Figure 2. The tensile strength of steel wires was 1860 MPa, the modulus of elasticity was 206 × 10^3^ MPa, and the linear expansion coefficient was 12 × 10^−6^/°C.

Ideally, given the effect of tight occlusion, all outer wires and inner carbon fiber smart bar are in a cooperative state. All longitudinal cross-sections of the composite cable follow the plane cross-section assumption, and strains of each strand in the same section are equal. Therefore, the longitudinal strain of the entire composite tendon is measured by the sensing elements of the middle smart bar. Fiber gratings are photowritten at specified locations in the fiber bonded to the smart rib to directly measure the strain in the structure. A plurality of fiber grating regions is also arranged in the optical fiber simultaneously. Thus, the longitudinal distribution of the strain along the same prestressed smart composite cable is simultaneously measured, and the laws of transmission and attenuation along the cable are obtained. This is suitable for understanding the mechanical properties and behaviors of prestressed structures and components and to obtain a set of strain monitoring information in a regular sense array to detect and identify the structural properties or types of structural damage by designing the location and number of the grating.

## 3. Experiment Design

As previously mentioned, the prestressed smart cable for concrete structures is a new type of functional prestressing tendon that is proposed to solve monitoring problems during construction and long-term service. To explore the mechanical properties and sensing performances of prestressed smart composite tendons and solve key technical bottlenecks in the engineering applications, the aim of the study involves examining their mechanical performance, the effect of the tensioning process on sensing performance, long-term holding capacity, and perception sensitivity for an additional live load during service by means of a tensile test, holding force test, and fracture test.

**Figure 3:**
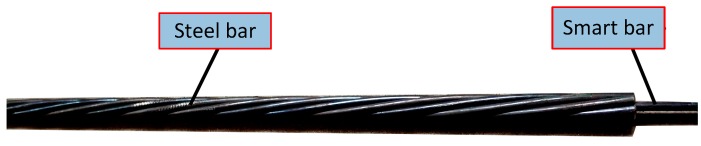
Photo of the prestressed smart composite cable (partial).

**Figure 4:**
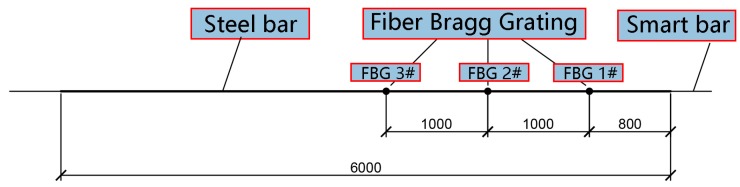
Distribution of the gratings for prestressed smart composite cable No. 1 and No. 2.

Based on the production process described above, three prestressed smart composite cables were produced wherein each was fabricated from six steel wires with a diameter of 5 mm and a smart bar with an identical size. Each wire was 6 m long. The photograph of the prestressed smart composite cable is shown in Figure 3. The length of both prestressed smart composite cable No. 1 and No. 2 corresponds to 6 m. Fiber gratings are placed at intervals of 800 mm, 1800 mm, and 2800 mm from the ends to measure strain at those locations. The layout is shown in Figure 4. Cable No. 3 is 6 m in length, and the FBG strain gauge is only set at a distance of 2800 mm from the end.

Three experiments were conducted. The first corresponds to the tensile test. The prestressed smart composite cable is subjected to repeated multi-stage tensioning by a jack, and it involves examining whether the mechanical and sensing performance can satisfy the requirements of prestressed tendons in prestressed concrete, and whether the embedded FBG sensor is suitable for use as a sensing device. The second experiment corresponds to the holding force test. A pre-tension force is continuously applied to the prestressed smart composite cable, and the sensing performance of the composite cable under long-term high stress state is examined. The third is a fracture test that examines the sensing ability to extreme destruction by continuously increasing the pretension exerted on the prestressed smart composite cable until it breaks. All three tests were loaded under stress control, and the control stress was 1302 MPa. Furthermore, the test conditions are identical to those of the ordinary prestressed tendons in the PC component in the service or in construction stage.

**Figure 5:**
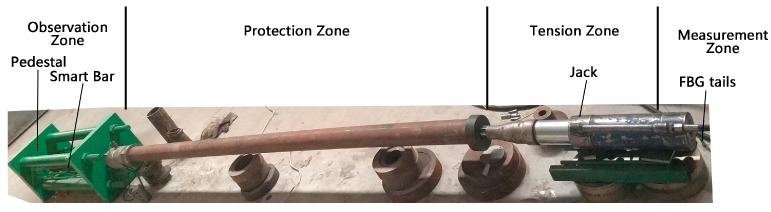
Experiment site layout.

The layout of the experimental site is shown in Figure 5. The site is divided into observation zone, protection zone, tension zone, and measurement zone. An end of the prestressed smart composite cable is fixed at the tension pedestal in the observation zone. The response of the cable is observed from the observation zone. The function of the protection zone involves protecting the prestressed smart composite cable with a steel pipe, ensuring safety, and maintaining tensile stress. The jack is located in the tension zone, and tensioning force is applied to the prestressed smart composite cable by using hydraulic equipment. In the measurement zone, the grating fiber of the prestressed composite smart cable is connected to a demodulator (GFR1002-CB, University of Science and Technology Beijing, Beijing, China) to obtain the monitoring data. The sampling frequency can be changed at will between 0.5 Hz–1000 Hz.

## 4. Test Results and Analysis

### 4.1. Tensile Test

**Figure 6:**
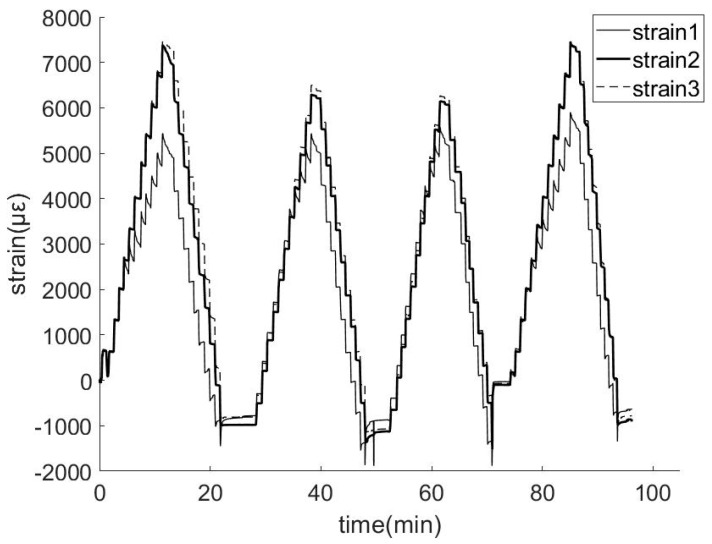
Three FBG strain gauge measurements in the tensile test.

The test was performed on a smart prestressed composite cable No. 1. In order to ensure the safety of the experiment, σ_m_ was set to 1302 MPa. And in order to simulate the tension process of ordinary prestressed tendons, the maximum control stress corresponding to 1.1 σ_m_ was divided into 11 levels. The tensile stress was gradually increased from 0 to 1.1 σ_m_ and then decreased to 0. Each level lasted for 1 min, and the gap between the two levels was also 1 min. Each loop took a total of 43 min. Each interval between loops was 5 min, and there was a total of four loops. During the tensioning process, the FBG demodulator continuously recorded the time and wavelength of FBG sensors, and then strain value was calculated. The sampling frequency was 40 Hz, and three sets of strain-time data were measured, as shown in Figure 6. In the figure, the horizontal axis denotes time in seconds, and the total time was approximately 100 min. The vertical axis denotes strain wherein the unit corresponds to με.

From Figure 6, the strain change in each sensor during four loading loops is clearly distinguished and specifically when the strain exceeds 5000 με, the sensor can still work normally. This indicates that the smart composite prestressed tendon exhibits a reliable sensing function during the loading process, and a plurality of sensors was used to simultaneously monitor multiple points in a composite cable.

The test results indicate the existence of certain differences in the data of three strain gauges in cable No. 1. The data for strain gauges 2# and 3# were almost identical, and both exceeded those of FBG strain gauge 1#. This was because the FBG strain gauge 1# was located near the end of the tendon, and the force transmission between the inner-layer smart bar and the outer steel wires was still unstable. However, the force transmission was completed at the position of FBG strain gauges 2# and 3# when the deformation of each section is coordinated, and it approximately obeyed the plane cross-section assumption. Therefore, the strain data at 2# and 3# is almost identical.

With respect to the same strain sensor under the identical tensile force, it was observed that the strains at the 2nd, 3rd, and 4th loadings and those at the unloading loops were almost equal although they significantly differed from the strain data from the first loop. This is because the mechanical setting between the smart bar and steel wires is not uniform along the longitudinal direction and the first loading and unloading loop can make the mechanical setting uniform. On the macroscopic level, it appears that the smart bar and the outer steel wires exhibit a certain degree of slip such that the strain data of first loop is excessively high. After the first loop, most of the slip is eliminated due to the first tension such that the strains at the same level of loading and unloading are almost identical. This indicates that in terms of practical engineering, pre-tensioning is essential to eliminate this part of the slip, and this is followed by the actual installation and use.

**Figure 7:**
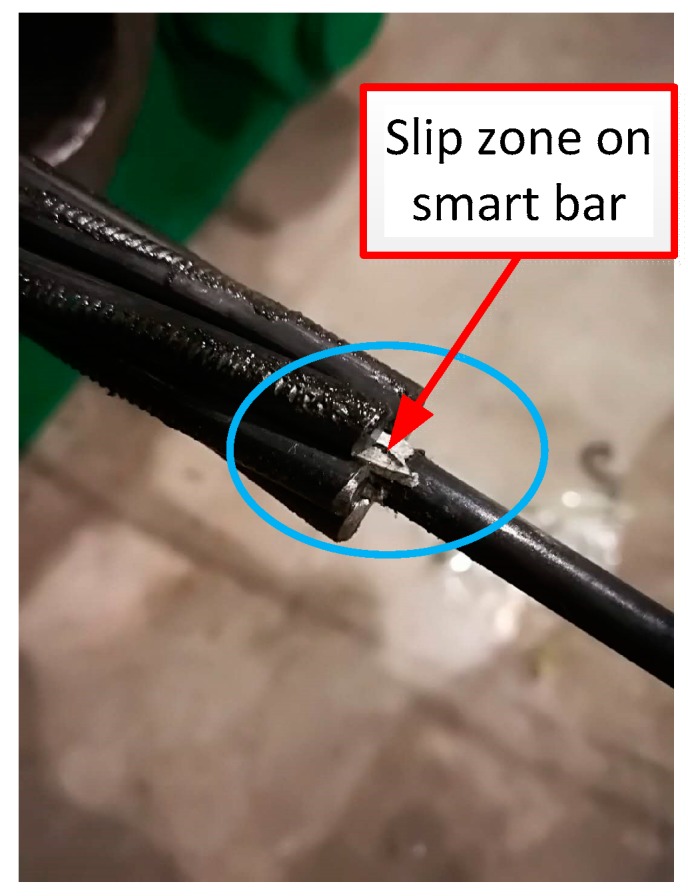
Photographs of the cable end after tensioning.

Additionally, it was also observed that the strains at a few levels corresponded to negative values at the 2nd and 3rd loops, and there was a partial strain residual at the end of the tension. This was also due to the lack of pre-tensioning. The slip and strain residual after tensioning were also verified by post-examination photographs (as shown in Figure 7).

**Figure 8:**
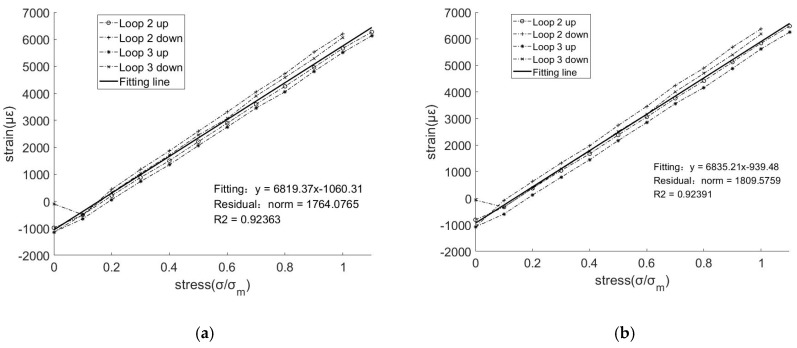
Sensing performance of the prestressed smart composite cables in the tension test: (**a**) Strain gauge 2# and (**b**) Strain gauge 3#.

The strain measurements of the 2nd and 3rd loops of FBG strain gauges 2 and 3 are considered as the vertical axis, and the control stress at each load level is considered as the horizontal axis to obtain the sensing performance curve of the two FBG gratings shown in Figure 8. It was observed that the strain linearity of the two FBG strain gauges during tensioning was extremely high, and the linearity was still good even under high deformation conditions exceeding 6000 με. Furthermore, the figure showed that the discreteness of the data was very small despite loading and unloading twice, and the fitting correlation coefficient exceeded 92%. This also indicates that the sensing performance of the smart composite prestressed tendon exhibits enhanced repeatability.

### 4.2. Holding Force Test

This test was performed on the prestressed smart composite cable No. 2. Based on test 1, it is necessary to eliminate the non-uniform connection between the inner smart bar and outer steel wires. Therefore, the cable was pre-tensioned such that the slip occurs to the maximum possible extent prior to the test. Subsequently, tension under the stress of 1.1 σ_m_ was maintained for 20 h. The sampling frequency was set to 2 Hz, and the strain data of all the FBG sensors during the holding force period were continuously recorded to check the cable’s retention of the mechanical properties and sensing performance.

**Figure 9:**
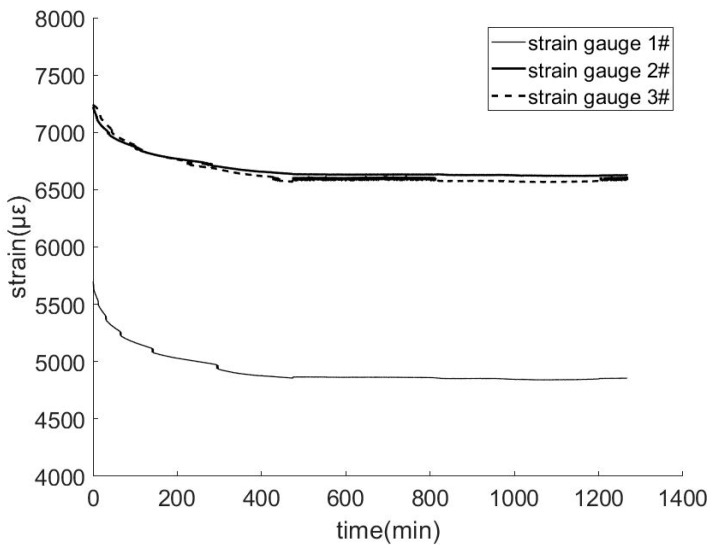
Long-term experiment for composite smart cable No. 2.

The strain data over the entire 20 h is shown in Figure 9, where the horizontal axis denotes time in hours and the vertical axis denotes strain with units in με. Three curves represent strain gauge 1#, 2#, and 3# in the figure.

As shown in Figure 9, during the complete process of tensioning, the three FBG strain gauges function properly, and abnormal data was not observed, thereby indicating that the sensing performance of the smart composite prestressed tendon exhibits high long-term stability. Additionally, the initial strain level in three strain-time curves were all extremely high, and this slowly decreased with time. After approximately 6–7 h, the strain tended to stabilize. This is because the oil path of hydraulic jack is unstable, and this is not related to the mechanical performance of the smart composite prestressed tendon. After the oil path was stable in the later period, the tensile force remained unchanged, and the data tended to be stable, thereby indicating that the smart composite prestressed tendon exhibits high long-term stress performance.

Furthermore, the strain data of the FBG strain gauge 1# was significantly lower than that of strain gauge 2# and 3#, and the data of strain gauge 2# and 3# were almost identical. This again illustrates the regularity of the force transmission of the internal steel wires and smart bar when it is subjected to tension, meaning the friction and occlusal forces transmit the tension from the outer wires to the inner smart bar. The force transmission was completed in the final region of the composite tendon. The deformation of each section in the middle part of the composite tendon is compatible and conforms to the plane cross-section assumption.

### 4.3. Fracture Test

An important goal of the development of a prestressed smart composite cable involves achieving the self-sensing of prestressed components in extremely destructive events by embedded smart sensing elements. Previous tests verified the mechanical properties and sensing performance of the composite cable during normal use, while the fracture test is used to simulate the performance of the prestressed concrete component by using the composite tendon under extreme load conditions. Thus, smart composite prestressed tendon No. 3 was used in the fracture test.

**Figure 10:**
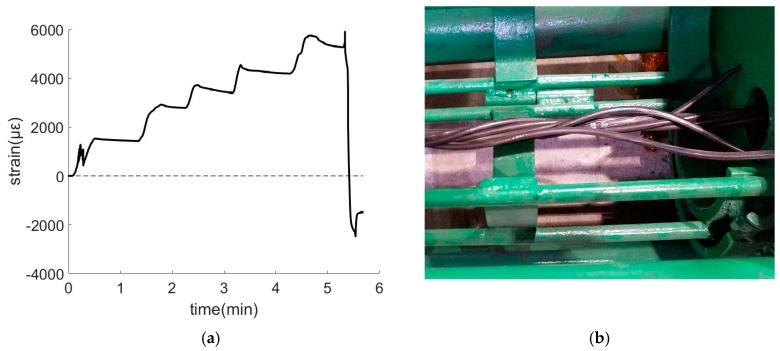
Sensing performance of prestressed smart composite cables in the fracture test: (**a**) strain-time curve in fracture test and (**b**) fracture form.

In the test, the tension started at zero then the load increased in steps until the composite tendon fractured. During the entire process, the FBG demodulator was used to continuously record the wavelength of the smart bar, and the sampling frequency was 40 Hz. The strain data recorded during tensioning is shown in Figure 10a. The horizontal axis denotes time with units in seconds. The length of the recorded data is approximately 6 min. As shown in the strain-time curve in Figure 10a, the smart composite prestressed tendon failed at approximately 370 s. At the time of fracture, it reached a strain of approximately 5900 με. The fracture occurred near the fixed end of the tensile pedestal. The main failure mode corresponds to when the outer steel wire is completely broken, as shown in Figure 10b.

During the tensioning process, the signal of the FBG sensor was not interrupted, and the FBG strain sensors worked continuously even after the composite cable was broke. The data obtained is in accordance with the basic laws of mechanics. This indicates that the smart composite prestressed tendon exhibits the ability to sense mechanical behavior over its entire life. It monitors stress under normal operating conditions, and also monitors stress in the process of damage and fracture of structures and components. Thus, the structural fracture process is accurately recorded.

## 5. Conclusions

To monitor the prestress state of a prestressed concrete structure, a smart composite prestressed tendon composed of a CFRP bar with steel wires wrapped around it was proposed that simultaneously performs load reinforcement and quasi-distributed stress monitoring. Experiments were performed to examine the mechanical performance and sensing performance of the composite cable during normal operation periods, and sensing performance under extreme conditions. The following conclusions were obtained:

1. The smart composite cable withstood an operating load with high strain. It worked stably under the tensioning process and relative long-term loading conditions of the construction stage. The cable was used as a prestressed tendon for prestressed concrete components, and it exhibited reliable work performance.
2. By embedding an FBG strain gauge on the inner carbon fiber-based bar of the smart cable, the self-sensing function of the stress state of the complete composite cable was effectively realized. The composite cable exhibited good linearity and repeatability, and it can be used to high pre-stress levels during normal operation. Additionally, it exhibited high long-term stability and displayed reliable sensing performance after failure.
3. The tension transmission of the composite cable requires a specific distance. Thus, consistent strain data was obtained when the FBG strain gauges were arranged in the area of deformation conformability beyond a certain distance from the end, and the measurement accuracy and reliability of the sensor simultaneously improved by arranging multiple FBG strain gages.
4. While using smart composite prestressed tendon as reinforcements, the slip phenomenon can occur during the first tensioning due to the non-uniform longitudinal connection between the inner carbon fiber based smart bar and outer steel wires. Recommendations include performing several pre-tensioning stages prior to construction, such that the slip occurs to the maximum possible extent in the pre-tensioning stage. Furthermore, the contact and engagement forces between the steel wires and smart bar during production should be increased to further improve the mechanical properties and sensing performance of prestressed smart composite cables.

Given the constraints of the conditions, the study involves a few limitations. First, the time of long-term holding test was not sufficient. Second, further studies are required to focus on the long-term durability, mechanical properties, and sensing performance of the smart composite cable under vibration and impact load and under fatigue loading conditions based on high working stress.
